# LARS&LISA: a universal school-based cognitive-behavioral program to prevent adolescent depression

**DOI:** 10.1186/s41155-018-0104-1

**Published:** 2018-08-29

**Authors:** Patrick Pössel, Eric Smith, Olivia Alexander

**Affiliations:** Department of Counseling and Human Development, University of Louisville, 2301 S. Third Street, Louisville, KY 40292 USA

**Keywords:** Prevention of adolescent depression, Cognitive-behavior therapy, School-based

## Abstract

Adolescent depression is a prevailing international mental health concern as up to 27% of adolescents experience either subsyndromal depression or a major depressive episode by the age of 18. Depression in adolescence has been found to negatively impact current and future academic achievement, functioning, mental health, and quality of life. Accordingly, the authors emphasize the importance of proactively preventing depression (and its negative outcomes) instead of waiting and having to “fix” the problems after they have already developed. The current article begins with a discussion of the various types of prevention, including their respective advantages and disadvantages. Further and more importantly, the article’s primary focus is to provide a summary of the theoretical basis, development of, empirical support for, and content of a universal school-based cognitive-behavioral program to prevent adolescent depression entitled LARS&LISA (*Lust An Realistischer Sicht & Leichtigkeit Im Sozialen Alltag*). As the program exists within the overlapping realms of universal prevention, school-based programs, and cognitive-behavioral interventions, the content of this article is relevant to all three areas and offers insight into the development of depression prevention in general. Finally, empirical support for the positive effects of the program is presented and some ideas for further research are discussed.

## Introduction

Through a systematic review of epidemiological studies on subsyndromal depression in adolescence, Bertha and Balázs (2013) found that rates of subsyndromal depression increase sharply around the mid-teen years and continue to rise through early adulthood. As a result of this increase, up to 27% of adolescents have a lifetime prevalence of either subsyndromal depression or major depressive episodes by the age of 18 (Bertha & Balázs, 2013; Kessler, Petukhova, Sampson, Zaslavsky, & Wittchen, 2012). Further, depressive symptoms are associated with a wide range of negative outcomes in adolescents (Bertha & Balázs, 2013; Kessler et al., 2012; Klein, Torpey, & Bufferd, 2008; Patel, Flisher, Hetrick, & McGorry, 2007). The high prevalence and multitude of negative consequences make subsyndromal and major depression in adolescents a serious international mental and public health concern. Thus, one of the aims of this article is to emphasize the need for preventative interventions to proactively curtail depression (and its negative outcomes) instead of waiting to treat symptoms after they have already developed. A second aim of the article is to describe the theoretical basis, development of, empirical support for, and content of a successfully evaluated universal school-based cognitive-behavioral program to prevent adolescent depression. Given that the program exists within the overlapping realms of universal prevention, school-based programs, and cognitive-behavioral interventions, the content of this article is relevant to all three areas and offers insight into the development of depression prevention in general.

## Depression and its consequences—why are we waiting?

According to a recent report, depression is now the leading cause of Years Lived with Disability (YLD), impacting an estimated 322 million people worldwide (World Health Organization [WHO], 2017). Adolescent depression is of particular concern (Patel, 2013) as it is estimated that between 22 and 27% of adolescents have experienced either subsyndromal depression or a major depressive episode by the age of 18 (Bertha & Balázs, 2013; Kessler, Petukhova, et al., 2012). Alarmingly, there has been an increasing trend in the prevalence of major depressive episodes among adolescents in recent years. In 2014, 11.3% of adolescents in the USA reported experiencing a major depressive episode within the previous year, compared to 8.7% in 2005 (Mojtabai, Olfson, & Han, 2016). Adolescent depression is associated with decreased quality of life, serious emotional disturbances, and poor to severe functional impairment (Bertha & Balázs, 2013; Kessler, Avenevoli, et al., 2012). Adolescents with depressive symptoms have been shown to have higher academic failure, delinquency, interpersonal distress, substance abuse, suicidality, and unemployment (Klein et al., 2008; Patel et al., 2007).

Further, adolescent subsyndromal depression and major depression are significant risk factors for subsequent depressive episodes (Bertha & Balázs, 2013; Johnson, Cohen, & Kasen, 2009; Kessler, Petukhova, et al., 2012; Klein, Shankman, Lewinsohn, & Seeley, 2009; Patel et al., 2007). Klein et al. (2009) reported that 67% of adolescents with subsyndromal depression developed major depression during a 15-year follow-up period and 27% of people with lifetime major depression experience their first episode in childhood or adolescence (Kessler, Petukhova, et al., 2012). In the best-case scenario, only 36% of the burden of depression could be alleviated using state-of-the art knowledge and therapies (Andrews, Issakidis, Sanderson, Corry, & Lapsley, 2004), but it is estimated that prevention programs have the potential to prevent an additional 21–22% of the incidence of depression (Cuijpers, van Straten, Smit, Mihalopoulos, & Beekman, 2008). This raises the question of why are we not embracing the wisdom contained in the idiom “an ounce of prevention is worth a pound of cure.”

## Types of prevention

Based on the evidence and considerations outlined above, effective programs that ameliorate currently existing depressive symptoms and prevent normative increases in depressive symptoms are needed (e.g., Horowitz & Garber, 2006). Prevention programs can be differentiated based on the target group they are designed for and are usually separated into three categories: indicated, selective, and universal. *Indicated* prevention programs are designed to help individuals with clear risk factors. *Selective* prevention programs are aimed at subpopulations known to be at increased risk for developing certain problems. These two types of prevention programs are often collectively categorized as *targeted* prevention. Finally, *universal* prevention is intended for all individuals, regardless of their risk for developing a problem. Each type of prevention has advantages and disadvantages, which are discussed in detail by Offord (2000) and Pössel, Schneider, and Seemann (2006). Offord lists many advantages (*n* = 6) and disadvantages (*n* = 8) for universal prevention but only two advantages and six disadvantages for targeted programs. As a detailed discussion of all advantages and disadvantages would be beyond the scope of this paper, only the most relevant points will be considered in the following.

One advantage of targeted programs is that they are generally more cost-effective than universal programs (Offord, 2000). Targeted prevention programs focus on disorders or behavioral issues that might only impact a small percentage of the entire population. Therefore, it is theoretically most efficient to only offer preventative interventions to this high-risk group, as opposed to all members of a given population. Additionally, targeted interventions can focus more intently on symptoms or risk factors that are experienced by every member in a group. However, targeted programs are not inherently the most cost-effective in every circumstance given the cost of screening for risk factors and the challenges associated with correctly identifying individuals who would benefit from such programs.

Not surprisingly, evaluation studies of targeted prevention programs usually find higher effect sizes than studies evaluating universal programs. The reasons for this are that studies on the former tend to have (a) participants with higher average levels of the target variables (e.g., depressive symptoms) and (b) a higher percentage of participants with elevated levels of symptoms than what is typical for participants in universal prevention programs. Furthermore, individuals in targeted prevention programs are generally more motivated to participate than the average person because of the distress being inflicted by the target problem, which may contribute to the comparatively larger effect sizes found for these programs (Offord, 2000; Pössel, Schneider, & Seemann, 2006).

One potential disadvantage of targeted prevention programs is that individuals may experience stigmatization based on their participation. Empirical literature indicates that adolescents may experience stigmatization from their family, teachers, and particularly their peers based on mental health issues (Moses, 2010; Platt, Kadosh, & Lau, 2013). A systematic review found that fear of stigma and embarrassment are primary barriers that prevent many adolescents from seeking mental health care (Gulliver, Griffiths, & Christensen, 2010). Therefore, it is clear that worries about stigmatization are a particularly salient concern for adolescents with regards to participating in an intervention. For prevention in a school setting, if only a small number of adolescents are selected to partake in a targeted prevention program, they may feel labeled and marginalized by their peers who were not recruited. The participants may experience not only negative subjective effects of perceived stigmatization, but also objective effects, such as a loss of social contacts (Moses, 2010). Further, Harrington and Clark (1998) speculate that the stigmatization that results from labeling might even lead to a chronification of depressive symptoms. In other words, it is possible that targeted prevention can actually have an iatrogenic effect on the individuals participating in such programs. While targeted prevention programs do not automatically lead to stigmatization of the participants, special attention must be given to ensuring that these programs do not lead participants to feel or actually be singled out or labeled.

While adolescents may fear stigmatization as a result of participating in targeted intervention programs, it seems that school administrators have their own reservations. Studies have found that school principals (Miller, Eckert, DuPaul, & White, 1999) and school psychologists (Eckert, Miller, DuPaul, & Riley-Tillman, 2003) have concerns about targeted programs. As a result, it may be especially challenging to acquire the necessary approval to implement targeted prevention in school settings. Horowitz, Garber, Ciesla, Young, and Mufson (2007) report that their attempt to implement a targeted depression prevention program was not approved by the school administration. Thus, they had to deliver their program to all students at the participating schools, turning it into a universal prevention program.

Another drawback associated with targeted prevention is that not all members of a high-risk group develop a problem (e.g., major depression) and most individuals that do develop a problem are not from a high-risk group (Offord, 2000). This issue reflects the difficulty in screening and accurately identifying which individuals will develop mental health problems, such as depression. One possible negative outcome of this issue is that individuals could be stigmatized for participating in a program that they do not actually need. The costs associated with screening a large number of adolescents, combined with the problems in accurately identifying which individuals would benefit from participation in a targeted prevention program call into question whether this type of program is truly the most efficient and efficacious form of prevention, particularly in school settings.

Although there are unique advantages and disadvantages associated with each type of prevention, some of the disadvantages of targeted prevention do not necessarily apply to universal prevention. For example, given that *all* students in a particular school or grade level participate in universal school-based programs, problems related to identifying and recruiting participants, and stigmatization may be mitigated. However, it is worth considering that requiring all students to participate in a prevention program could have the unintended effect of having those who need the intervention most feel marginalized by putting them in a group of students who are not experiencing the same symptoms. Students experiencing a particular problem, such as depression, may feel reluctant to share their experiences and participate in the group if they feel they are the only ones dealing with that problem. In an evaluative study of a school-based universal depression program in the Netherlands, Tak, Lichtwarck-Aschoff, Gillham, Zundert, and Engels (2016) hypothesized that the group climate may have contributed to their finding that the program was not effective for adolescents with high baseline depressive symptoms.

While it is important to acknowledge that there is still a potential for stigmatization associated with universal prevention programs, these programs have an advantage over targeted programs in that their intended purpose may be less outwardly apparent. For example, in the below described universal prevention program, LARS&LISA, the trained skills are framed as helping the adolescents to reach their personal goals. As a result, students’ apprehensions related to the stigma associated with receiving mental health care can be quelled. Further, while the programs focus on reducing cognitive patterns and behaviors that contribute to the development of depression, it is not necessary for students to disclose information that would make them personally vulnerable to benefit from the program. Although there is still a potential for some students to feel marginalized in universal prevention programs, the risk is diminished.

Another advantage of universal prevention programs is that the inclusion of all individuals in a particular group makes it likely that some group members already have the skills that the program focuses on. These group members can serve as peer role models for the members who would benefit from improving such skills (Harrington & Clark, 1998; Lowry-Webster, Barrett, & Dadds, 2001). Further, by participating in universal prevention programs, even individuals who are not at risk of developing the targeted problem benefit from the skills trained in these programs (Harrington & Clark, 1998). Building on this argument, Offord (2000) argued that while the effects of targeted programs are bigger on individual participants, universal programs can still have enormous effects on a system (e.g., a school or society) as they have (smaller) effects on a significantly larger number of individuals.

## LARS&LISA—the theoretical foundation

For the reasons described above, universal prevention seems to be a beneficial type of prevention, particularly for implementing in school settings. A majority of the currently existing universal depression prevention programs apply cognitive-behavioral methods and focus on adolescents (Bastounis, Callaghan, Banerjee, & Michail, 2016; Clarke, Hawkins, Murphy, & Sheeber, 1993; Merry, McDowell, Wild, Bir, & Cunliffe, 2004; Sawyer et al., 2010; Shochet et al., 2001; Spence, Sheffield, & Donovan, 2003, 2005). This article presents LARS&LISA (Pössel, Horn, Seemann, & Hautzinger, 2004), a universal school-based prevention program that is supported by research produced over the past two decades. LARS&LISA uses cognitive-behavioral strategies and is informed by Dodge’s social information processing model (Dodge, 1993). Table 1 summarizes key details about the program and research examining its effects.

**Table 1:** Overview of LARS&LISA intervention

1. Brief name	LARS&LISA—Lust An Realistischer Sicht & Leichtigkeit Im Sozialen Alltag
2. Why (rationale)	The primary aim of this universal, school-based prevention program is to prevent the development or increase of depressive symptoms among adolescents. Depression is a leading mental health concern for adolescents and is associated with a wide array of negative outcomes, which highlights the need for preventative interventions tailored to this population. The theoretical foundation for the program is Dodge’s (1993) social information processing model. Following this conceptual framework, the negatively biased way in which some individuals perceive, interpret, and respond to environmental stimuli contributes to the development of depression. Cognitive-behavioral interventions contained within the program are used to teach adolescents how to identify maladaptive thought patterns and behaviors and replace them with more helpful and realistic alternatives.
3. What (materials)	LARS&LISA is a structured program with a manual that provides detailed instructions, examples, and tips for group leaders on how to administer the intervention. The primary materials utilized in the implementation of LARS&LISA are handouts and worksheets, which aid the adolescents in understanding and remembering elements of the program’s content. For example, “Knowledge Checks” are worksheets that the students complete individually at the end of sessions, which are designed to reinforce the concepts that were covered that day. These worksheets simultaneously provide group leaders with feedback about the students’ comprehension of the material. Poster sheets, a whiteboard or blackboard, and a projector can be employed by the group leaders as visual aids to present guidelines, concepts, examples, etc. Candy or popular snacks can be used to create a motivational reward system that encourages participation and observation of the group’s guidelines. Finally, a few specific games and activities require additional materials. For instance, in the initial session, the participants compete in a relay race that requires plastic cups, straws, and small candy-coated chocolates. The LARS&LISA manual can be requested by contacting the authors of the program.
4. What (procedures)	Each session of the program follows a similar structure and contains a number of common elements. Group leaders begin by presenting the agenda for the day, providing feedback about last session’s Knowledge Check (described above), and asking how the students might have used the skills taught in LARS&LISA since the last session. A majority of the session is spent on group activities related to one of the five modules (Set Some Goals, Reversible Spiral, Think Tank, Just Do It, and Making Contact). Within these modules, students learn how to generate useful personal goals; understand the interconnected nature of thoughts, feelings, and behaviors; and replace maladaptive thoughts and behaviors with more helpful and realistic alternatives. Group activities may include interactive conversations, the completion of worksheets, or the creation and acting out of role-plays, depending on the session. Knowledge Checks are completed towards the end of most sessions. The group leaders end each session by making connections between that day’s content and the content presented in previous sessions, providing feedback about the observation of guidelines, and giving a preview of the next week’s session.
5. Who provided	The intervention has been successfully implemented by psychologists and supervised students in graduate psychology programs. A study was conducted to evaluate whether the program could be implemented by teachers, but like other similar programs, teachers were found to be less effective as group leaders.
6. How (mode of delivery)	The program is delivered in person in a group setting. The ideal number of participants is between 8 and 12, but the program has been implemented with larger (*n* ≤ 20) and smaller (*n* = 4) groups. It is recommended that two leaders co-lead a group, but groups have also been led by individuals and triads.
7. Where	LARS&LISA is designed to be a school-based intervention and therefore is generally administered in classrooms during school hours.
8. When and how much	The program was originally designed to be presented in 10, 90-min weekly sessions. However, the material has been reorganized to accommodate the unique class schedules at various schools that have hosted the program. For example, it has been adapted to be delivered in 16, 60-min weekly sessions for a school that had shorter class periods. The groups are always gender homogenous.
9. Tailoring	Given that each group presents a unique dynamic, minor accommodations are sometimes necessary to meet the specific needs of individual groups. For example, some groups tend to prefer more group work whereas others prefer to process the material individually. Additionally, some groups respond the best when leaders present content in a didactic manner, while others benefit more from game-like and interactive delivery methods. The program’s manual contains a number of suggestions for alternative ways to deliver the content and group leaders are encouraged to make adjustments to best meet the needs of each group.
10. Modifications	LARS&LISA has been modified a number of times to make improvements and accommodate various populations. The program originally contained four main modules, but a fifth was added to increase motivation and make the content more personally relevant to adolescents without depressive symptoms. Additionally, changes were made to make the content more relatable for male adolescents. Finally, the program was originally developed in German and was later translated into American English and culturally adapted to be implemented in the USA.
11. How well (planned)	To assure adherence to the manual, group leaders participate in a two-step training and while they implement the program in weekly supervision sessions using video recordings of each session. During the first step of the training, future group leaders participated in a mock version of the program, and in the second step, they study the manual, materials, and procedures and discuss their questions with their supervisors.
12. How well (actual)	This has not yet been addressed.

**Fig. 1:**
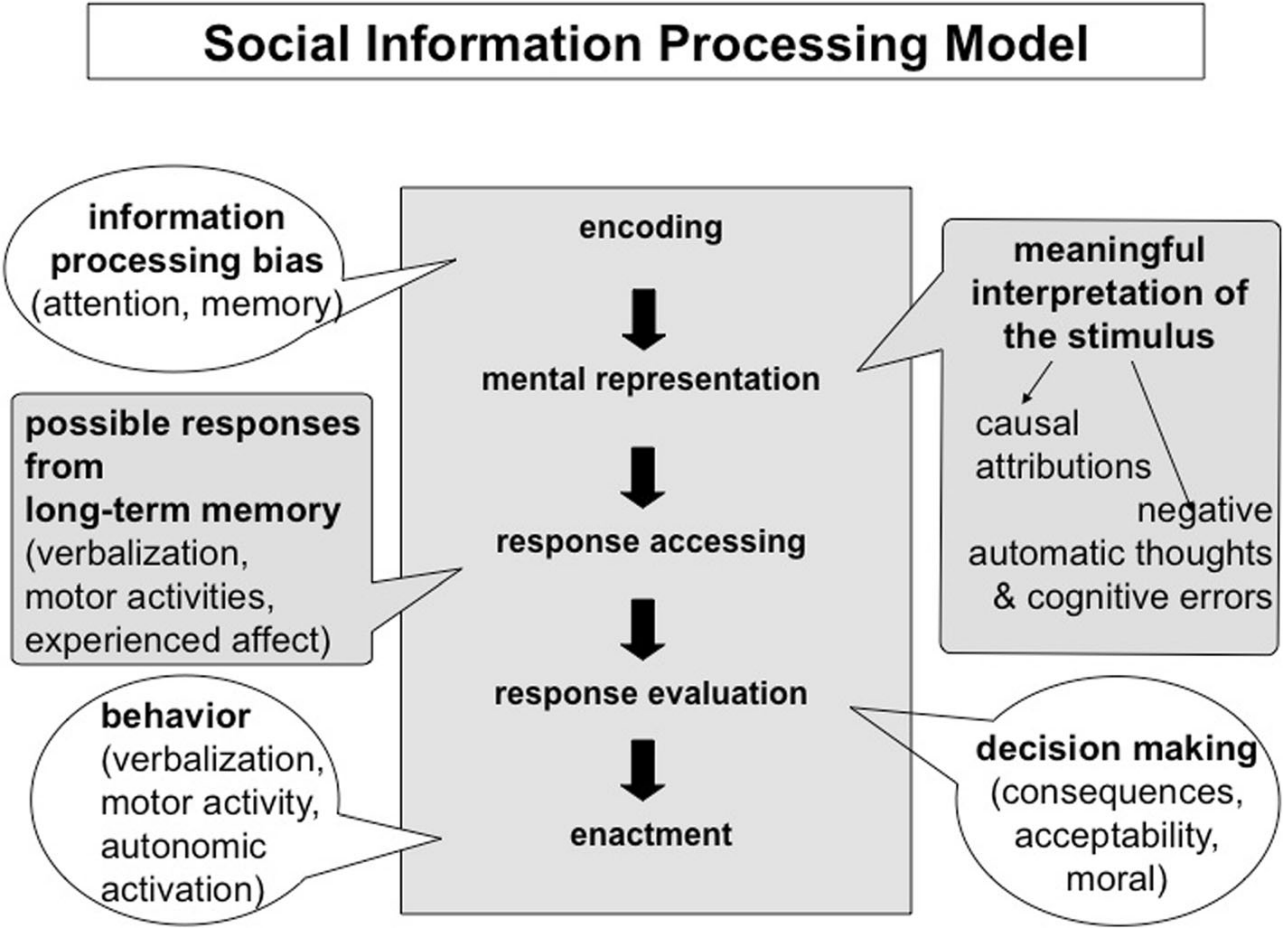
Dodge’s Social Information Processing Model (1993)

While there are a multitude of models describing the development and maintenance of depression, only a few of these models originated from a developmental psychopathology perspective. One such model is Dodge’s social information processing model (1993), which in its first iteration describes the development and maintenance of both depression and aggression. In this model, behavior is viewed as the product of a sequence of information processing steps. This sequence, which is precipitated by a situational stimulus, can be seen as a repeating, conscious, or unconscious process in social interactions. The social information processing model comprises five stages (Fig. 1).

First, in the *encoding stage*, relevant aspects of stimuli are detected by a selective perception. In everyday life, individuals are continuously being exposed to a multitude of sensory input. To manage and make sense of this input, individuals learn to selectively attenuate to the features of their environment that they deem to be the most relevant, which are then encoded into short-term memory. Adolescents who become depressed tend to have an information processing bias which leads them to select aspects of a stimulus that are consistent with their negative self-schema (Beck, 1976). In the following *mental representation* stage, individuals attribute meaning to the aspects of stimuli that they have encoded and stored in their short-term memory. For instance, if an individual experiences a negative event, they would ascribe a reason as to why that event occurred. The subjective determination of meaning depends on a person’s attribution style as well as other factors (Abramson, Alloy, & Metalsky, 1989). In this second stage, depressed adolescents tend to interpret negative stimuli as global and stable. Each interpretation is associated with multiple possible reactions, including affective change, arousal of the autonomic systems, endocrine secretion, physical activity, and verbalization. One or more of these possible reactions are initiated during the *response accessing* stage of information processing. While some responses occur automatically, such as arousal of the autonomic nervous system, individuals can elect to enact or withhold possible physical and verbal responses. In the *response evaluation and selection* stage, the processing individual evaluates the prepared reactions on the basis of acceptability, anticipated consequences, and/or moral values. Depressed adolescents tend to see social withdrawal as the least negative response. If a prepared reaction fulfills the evaluation criteria, it will be initiated in the *enactment* stage.

Although presented separately, the stages of information processing often occur simultaneously. Therefore, adolescents process new information while they select responses for and react to stimuli they were exposed to earlier. However, like with all stage models, Dodge (1993) proposes that the pathway from a stimulus to the corresponding response is linear and the processing steps for each stimulus follow the outlined sequential order. While the most recent version of the social information processing model focuses on aggressive behavior, the iteration of the model presented here has been supported by several studies regarding depression (e.g., Harrist, Zaia, Bates, Dodge, & Pettit, 1997; Pössel, Seemann, Ahrens, & Hautzinger, 2006).

## LISA, LARS&LISA, and TIM&SARA—20 years of change and consistency

### History and development of the program

This program was first developed almost 20 years ago in Germany under the name LISA (Leichtigkeit Im Sozialen Alltag; Pössel, Baldus, Horn, Groen, & Hautzinger, 2005) and underwent multiple revisions (LARS&LISA; Lust An Realistischer Sicht & Leichtigkeit Im Sozialen Alltag; Pössel, Horn, Seemann, & Hautzinger, 2004) and cultural adaptations (e.g., USA: TIM&SARA, Together Initiating More Socially Advantageous & Realistic Attitudes; Pössel, Martin, Garber, & Hautzinger, 2013) since then. All versions of the program are implemented in gender-homogenous groups. The reason for this is that research indicates that gender-homogeneous groups can create spaces where young people, and particularly boys, can share their emotions and feelings without embarrassment (Sukhnandan, Lee, & Kelleher, 2000). They are also likely to be less distracted, more open and responsive, and can participate without the fear of compromising their image in front of girls (Warrington & Younger, 2003). Further, the authors report that a pilot study revealed that adolescents worked more effectively and openly as a team if no peer from the other gender was in the room (Pössel, Horn, Seemann, & Hautzinger, 2004).

Originally, LISA included four main modules:

- Reversible Spiral (associations among thoughts, feelings, and behavior),
- Think Tank (identification of dysfunctional thoughts, reality check on dysfunctional thoughts, development of functional thoughts, rehearsal of functional thought process),
- Just Do It (assertiveness training)
- Making Contact (social competence training)

After the first evaluation studies (Groen, Pössel, Al-Wiswasi, & Petermann, 2003; Pössel et al., 2005; Pössel, Horn, Groen, & Hautzinger, 2004; Pössel, Horn, & Hautzinger, 2006; Pössel, Seemann, & Hautzinger, 2008), the authors integrated the character LARS into the program to provide a male main character with whom the male students can identify. In addition to being an acronym for the title of the program, the names LARS and LISA (or TIM and SARA in the American version) also represent two fictional characters that are used to present some of the program’s content. As an example, LARS/TIM is one of the characters in scripted role-plays used to model specific types of behavior to male students, and LISA/SARA is featured in the role-plays used with female students. Additionally, the fifth module “Set Some Goals” in which the participants develop personal goals was added while the content of the other four modules was streamlined to keep the program limited to 10, 90-min weekly sessions (Table 2). Given that the intervention is implemented in school settings, adjustments have been made to the number and duration of sessions to accommodate class schedules at certain hosting schools. For example, the program was adapted to be delivered in 16, 60 min at a school that had shorter class periods. Further, LARS&LISA was translated into American English and modified for youth in the USA where it is implemented under the name TIM&SARA. However, publications regarding the American version of the program still use the name LARS&LISA. The modifications for American adolescents primarily included the construction of culturally appropriate role-plays in which relevant idiomatic expressions were used (Pössel et al., 2013). While they are not reported in this article, it is worth noting that LARS&LISA has served as a model for the creation of prevention programs in other countries. LARS&LISA was one of the templates used by researchers in Chile (Araya et al., 2014) and Colombia (Gómez, Jimenez, & Restrepo, 2004) to develop similar programs that accommodate their cultural backgrounds and language.

**Table 2:** Content of LARS&LISA

Session	Topic	Objectives	Content
1	Introductions, Build Relationships	Explain guidelines; create a cooperative atmosphere; provide overview and rationale for topics in the program	- Get to know each other - Establish basic guidelines: fairness, respect, teamwork, and a positive working atmosphere - Consequences for breaking of guidelines - Introduce program
2	Setting Goals	Identify and develop goals	- Define goals - Setting realistic and achievable personal goals
3	Reversible Spiral-I	Learn connections among feelings, thoughts, and behaviors; teach concepts of “down” and “up” thoughts	- Define “feelings,” “thoughts,” and “behavior” - Reversible Spiral: associations among feelings, thoughts, and behaviors - Introduce “down thoughts” (self-critical, action-blocking) and “up-thoughts” (self-supportive, helpful)
4	Reversible Spiral-II	Identify self-critical, action-blocking thoughts	- Experience the Reversible Spiral - Explore meaning of negative thoughts
5	Think Tank-I	Question self-critical, action-blocking thoughts; generate self-supportive, helpful, realistic thoughts	- Introduce the “reality check” - Create one’s own counter thoughts (i.e., realistic “up-thoughts”)
6	Think Tank-II	Learn why self-supportive, realistic thoughts can be important and how to integrate them into one’s life	- Review meaning of and rationale for “up thoughts” - Identify daily situations in which “up thoughts” can be integrated
7	Just Do It-I	Learn differences among assertive, passive, and aggressive behaviors and their consequences. Review connections between thoughts and behavior	- Identify signs of assertive, passive, and aggressive behavior - Discuss pros and cons of different behaviors - Explore associations among negative thoughts; counter thoughts; and assertive, passive, and aggressive behaviors
8	Just Do It-II	Practice assertive behavior Practice not avoiding	- Demonstrate assertive behaviors - Practice assertive behavior in role-plays
9	Making Contact-I	Learn how to build and maintain friendships	- Demonstrate verbal and nonverbal strategies to signal interest in others - Role-play “making contact”
10	Making Contact-II	Practice building friendships. Obtain feedback about the program	- Participants evaluate and provide feedback - Hand out certificates of program completion - Good-byes and celebration

### Theoretical foundation of LARS&LISA

The first module of LARS&LISA is “Set Some Goals,” which is based on an approach developed by Kanfer, Reinecker, and Schmelzer (1996). As described above, this module was added later to help increase the motivation of the adolescents to actively participate. Adolescents develop personal goals, or at least share ones they already have, and learn what they can do in the present to reach these goals. According to Locke and Latham (1990), goals should be specific, concrete, and divisible into subgoals so that progress is measurable. The group leaders of LARS&LISA point out at the beginning of each module how the content and skills covered in the ensuing sessions can help the adolescents to reach their personal goals. This serves to motivate adolescents who have no depressive symptoms, focus the participants on something positive as opposed to just talking about problems, and connect all of the modules with each other.

As mentioned above, the theoretical framework of this school-based cognitive-behavioral depression prevention program is grounded in Dodge’s social information processing model (Dodge, 1993). The goal of LARS&LISA is to work on all stages of social information processing with the exception of the *encoding* stage, as there are currently no known intervention strategies targeting this stage. As described above, the interpretation of social stimuli takes place in the *mental representation* stage and the adolescents work on their interpretations by identifying dysfunctional, automatic thoughts (Beck, 1976) and replacing them with more functional thoughts (as introduced by the “Think Tank” modules). In order to develop an understanding of the key concepts and a motivation for change, the group members explore the relationships among cognitions, emotions, and behavior (as introduced by the Reversible Spiral module). During the *response accessing* stage, different reactions to social stimuli are activated or generated. These responses are then judged based on their potential consequences and acceptability during the *response evaluation and selection* stage. Finally, one of the response options considered in the third and fourth stages of the model is implemented in the *enactment* stage. Problems encountered by depressed adolescents during the *response accessing* stage result in the production of multiple problem-irrelevant responses (Mullins, Siegal, & Hodges, 1985) and only a few functional responses (Frye & Goodman, 2000). In addition, while depressed adolescents report more negative and less positive expectancies of withdrawing behavior than their non-depressed peers, they evaluate withdrawing behavior as more positive during the *response evaluation and selection* (Garber, Quiggle, Panak, & Dodge, 1991). These problems are targeted within LARS&LISA by the conscious generation of functional responses and the demonstration of the positive consequences of those new responses. Thus, to work on those problems, the program applies widely used effective methods of social competence trainings. In particular, the self-assertiveness (Just Do It) and social competence (Making Contact) modules provide training in behavior that is not compatible with social withdrawal. Within these modules, role-playing provides the main method of change as they allow for the simultaneous restructuring in both stages of information processing. During role-plays, adolescents can experiment with new, more functional response alternatives and observe their own and others’ reactions to these responses. At the same time, they learn to translate the new response options into actual behavior.

**Fig. 2:**
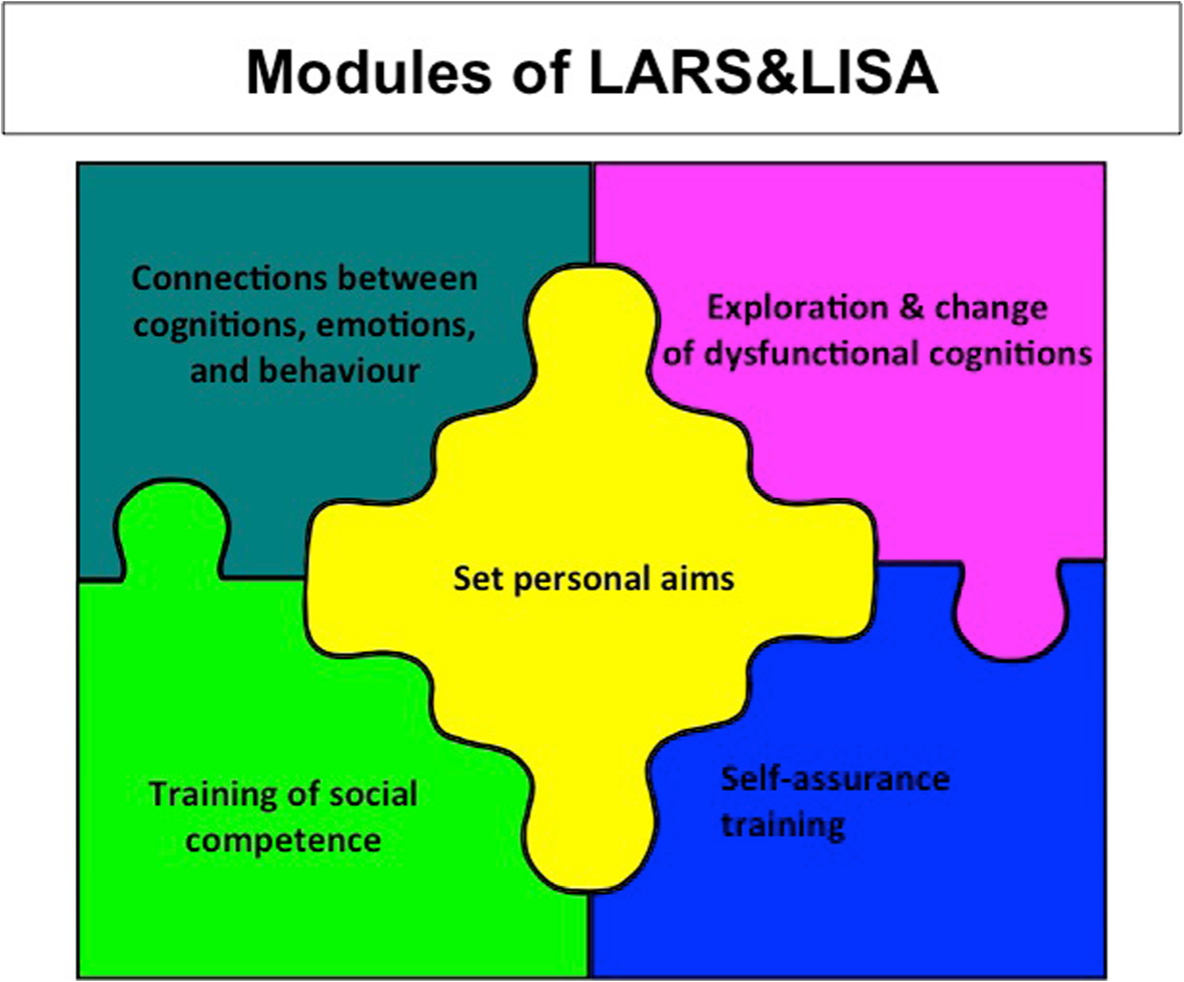
Modules of LARS&LISA

Summarized, the five distinct modules of LARS&LISA (Fig. 2) apply different standard cognitive-behavioral methods. Thus, the findings presented below are not only relevant for this particular program, but can also be seen as relevant for cognitive-behavioral depression prevention programs for adolescents in general. This is crucial as most adolescent depression prevention programs that are currently in existence use cognitive-behavioral methods (Bastounis et al., 2016; Clarke et al., 1993; Merry et al., 2004; Sawyer et al., 2010; Shochet et al., 2001; Spence et al., 2003, 2005).

### Specific content within the five modules

#### Module 1: “Set Some Goals”

**Fig. 3:**
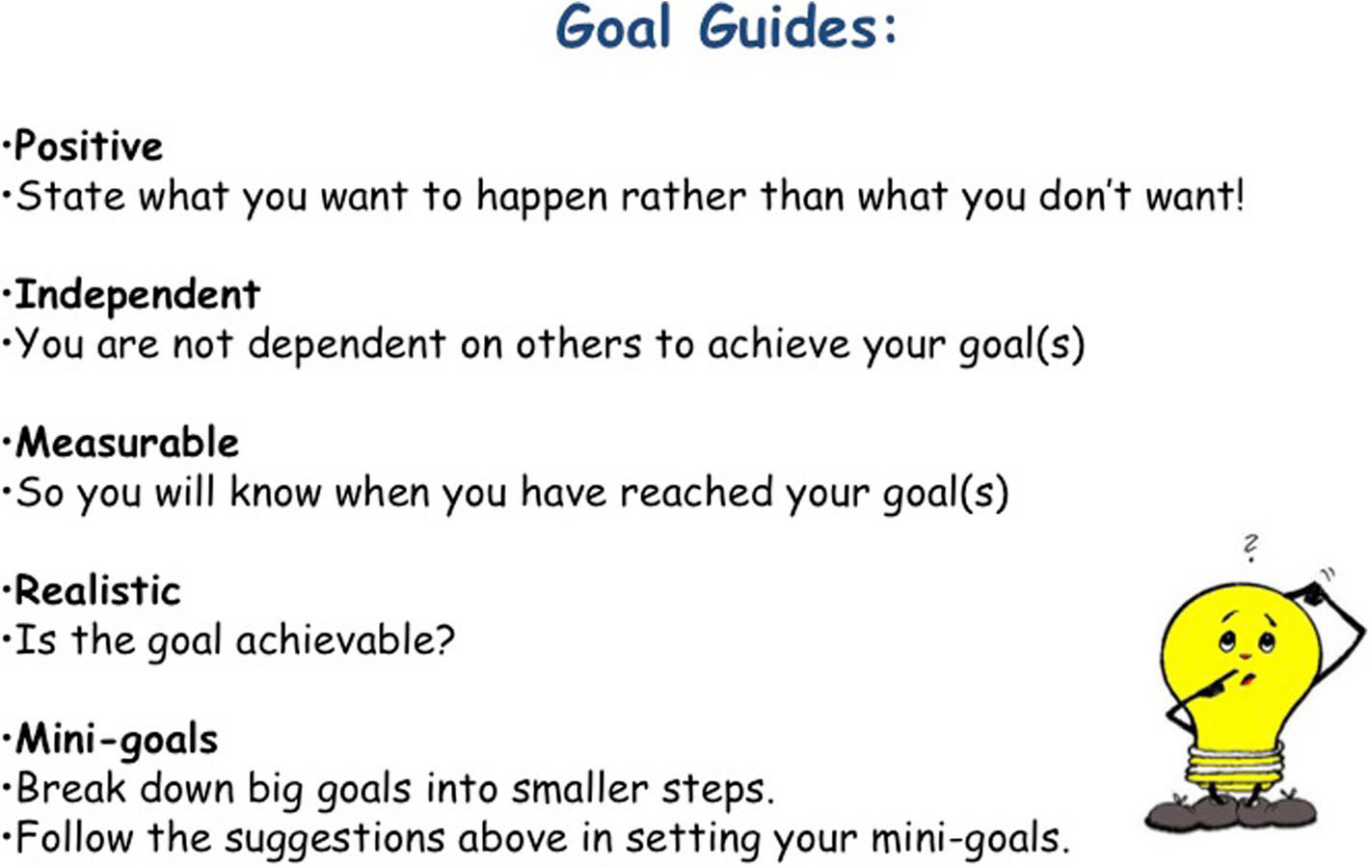
Goal Guides

**Fig. 4:**
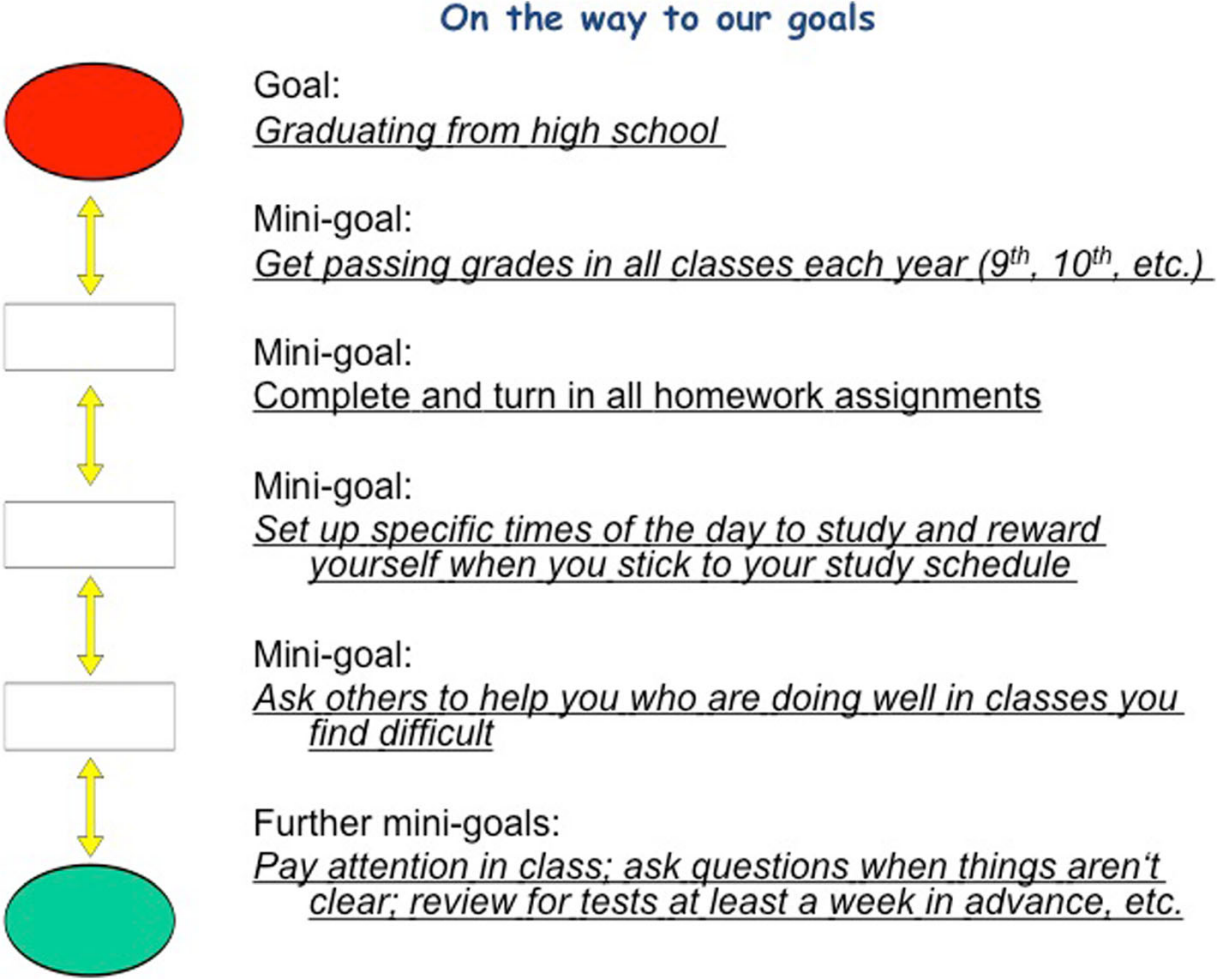
Example of a completed worksheet used to practice forming mini-goals

In accordance with the intended purpose of the “Set Some Goals” module, group leaders help students to develop goals that are individually meaningful, motivational, and realistically attainable. Before the students develop their own personal goals, they are presented with five “Goal Guides” for making helpful goals. The students are taught that their goals should be *positive* (things that the students hope to bring about as opposed to things they hope to avoid), *independent* (not reliant on someone else’s actions to achieve), *measurable* (operationalized in such a way that progress is easily identifiable), *realistic* (something than can feasibly be accomplished), and divisible into smaller, more manageable *mini-goals*. The handout that the students receive outlining these goal guides can be seen in Fig. 3. Examples are used to help the students fully grasp the five guidelines. For instance, leaders ask the group, “which of the following two goals is more measurable: ‘I want to get an A on the next math test’ or ‘I want to do better in school?’” Additionally, students have the opportunity to practice breaking large goals into smaller mini goals. A completed example of a worksheet used to practice forming mini-goals is shown in Fig. 4. Most activities are completed at least once as a group, and then, the students are able to practice the newly learned skills individually on worksheets. Interspersing group activities with individual work allows the group leaders to offer additional instruction to specific students as needed.

Following the introduction of the goal-setting guidelines, group leaders help the students to form personal goals, which are used to contextualize the material presented throughout the rest of the program. It is important for the students’ goals to be well developed, as these goals are used to show how the skills taught throughout the program are salient and useful for everyone. Students write down several personal goals as the group leaders circulate and ensure that the goals follow all of the guidelines. Each student indicates one goal that he or she is willing to display anonymously to the rest of the group. Prior to the next meeting, the group leaders write each signified goal on a paper leaf and attach the leaves to a large poster with a drawing of a tree. This “Goal Tree” poster is displayed during all of the subsequent sessions so that leaders can refer back to specific goals and discuss how LARS&LISA skills might be useful in accomplishing that goal.

Another important aspect of the “Set Some Goals” module is a discussion about potential barriers to accomplishing goals. Preemptively identifying possible obstacles helps students to anticipate challenges they will encounter as they pursue their goals. Addressing these potential barriers ahead of time allows the students to develop strategies for coping with challenges as they arise.

#### Module 2: “Reversible Spiral”

The objective of the second module is to elucidate how thoughts, feelings, and behaviors are all interconnected and influence each other. Students are taught to recognize the automatic thoughts that are triggered by stressful situations and how these thoughts might impact how they feel or behave. As a first step, the students are taught to differentiate between thoughts and feelings. They are presented with the following strategy: “If we can question or test it, then it is probably a thought. There is no way to test the truth of a feeling.” To practice distinguishing thoughts from feelings, the students are presented with examples and asked to identify them as either a thought or a feeling. One such example found on a worksheet used in this module reads, “Lars/Tim thinks he has really goofy hair that makes him kind of depressed about the way he looks.” Skills that are taught later in the program rely on the students’ ability to know the difference between thoughts and feelings and identify certain types of thoughts.

**Fig. 5:**
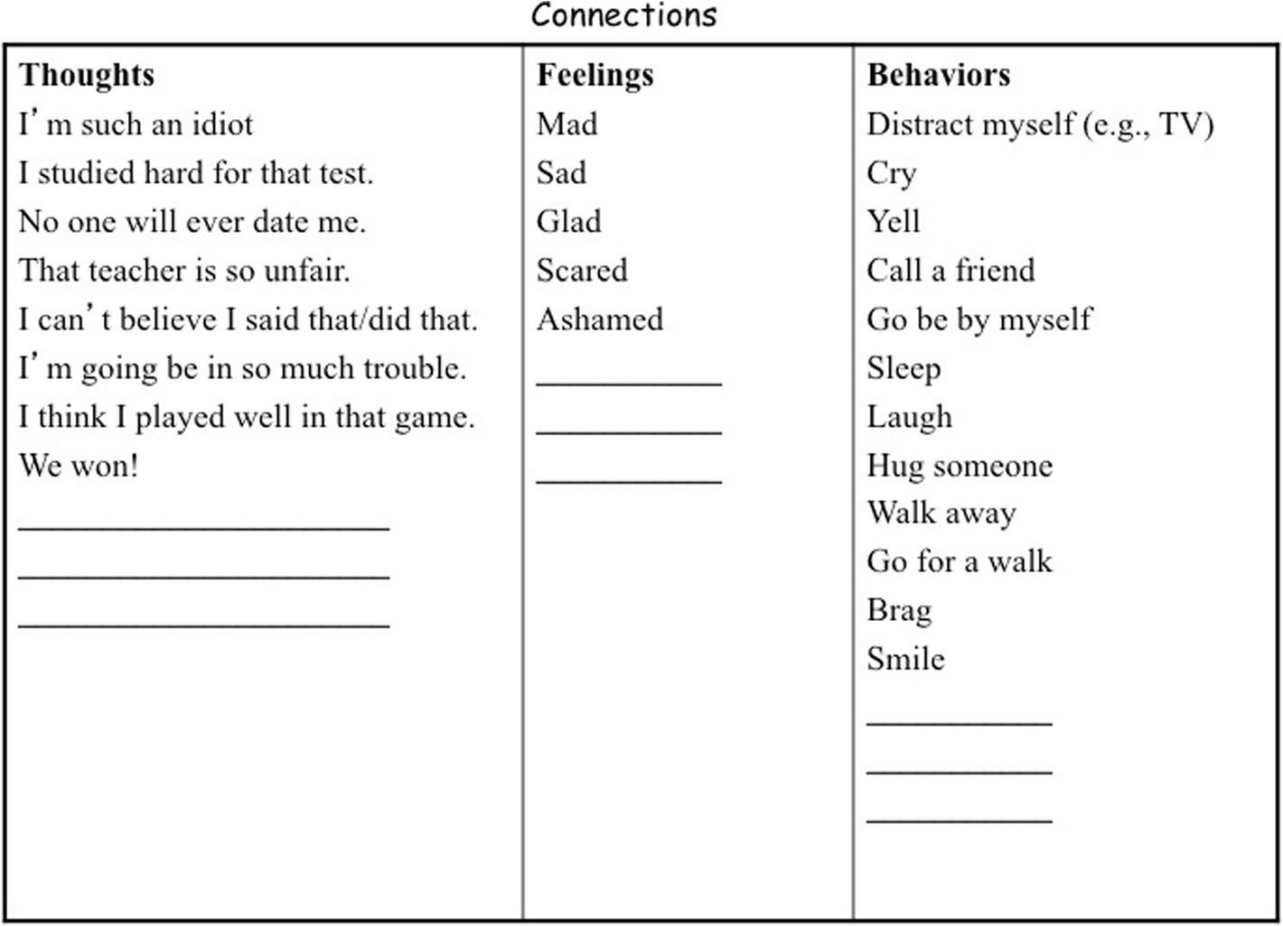
Connections game

**Fig. 6:**
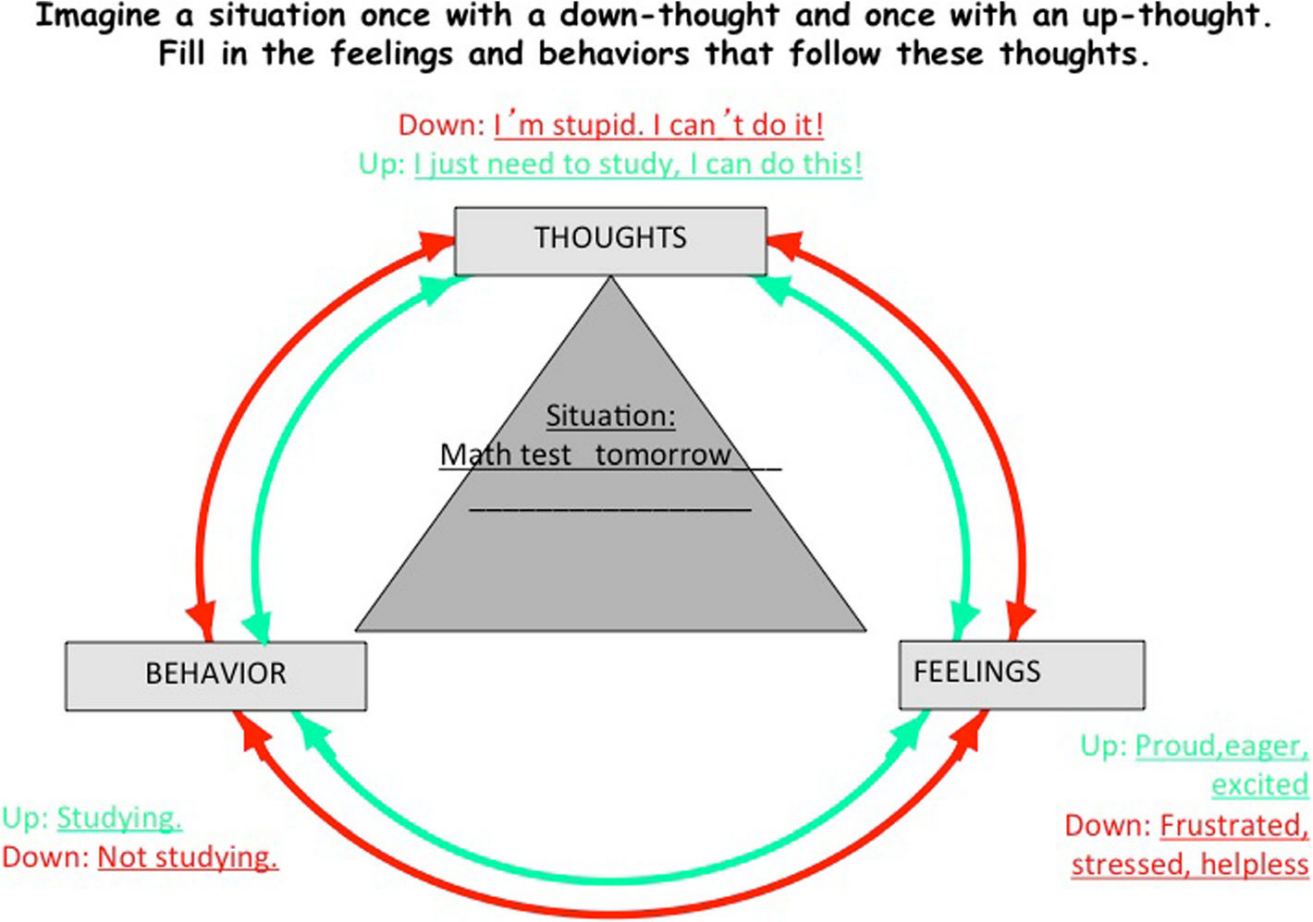
Example for the Reversible Spiral

A number of activities are used to demonstrate the relation between thoughts, feelings, and behaviors in the “Reversible Spiral” module. In one activity, the connections game, the students are presented with separate lists of thoughts, feelings, and behaviors (Fig. 5). Each student is asked to connect one thought from the list to a corresponding feeling and behavior. The goal of this activity is to demonstrate how thoughts, feelings, and behaviors are associated and how different situations can result in very different feelings and behaviors, depending on the thoughts a person has. The “Reversible Spiral,” which is depicted visually on posters and worksheets (Fig. 6), also serves to illustrate the interrelated nature of thoughts, feelings, and behaviors. Students are introduced to “up-thoughts” (thoughts that are realistic and helpful) and “down-thoughts” (thoughts that are not helpful or realistic and/or have an action blocking effect). The group is asked to come up with down-thoughts that might spawn from a given situation. This module focuses primarily on identifying down-thoughts, while the next module teaches students to actively replace down-thoughts with up-thoughts.

Down-thoughts are broken down into four distinct categories, which are intended to make down-thoughts easier to recognize and identify. *Demanding* down-thoughts involve thinking that things *must* or *have to* be a certain way. *Generalizing* down-thoughts are thoughts that are too extreme or all-encompassing to be realistic. *Worst-case scenario* down-thoughts involve adopting an overly negative viewpoint, such as thinking something is totally *horrible* or *terrible*. Finally, *making mountains out of molehills* is when a small piece of evidence is used to make a generalized, largely unsupported inference. One way to familiarize group members with the four types of down-thoughts is to split the students into two teams and play the “Down-Thought Game.” In this game, group leaders read out examples of down-thoughts, such as, “No one will ever think I’m cool.” The two teams compete to try and correctly identify which category the down-thought would fall into (in this case the correct answer would be *generalizing based on the keywords “no one” and “ever”*). The idea behind identifying down-thoughts is that it aids the students in recognizing their own negatively biased automatic thoughts. This recognition is the first step in replacing maladaptive thinking patterns with more helpful alternatives.

#### Module 3: “Think Tank”

**Fig. 7:**
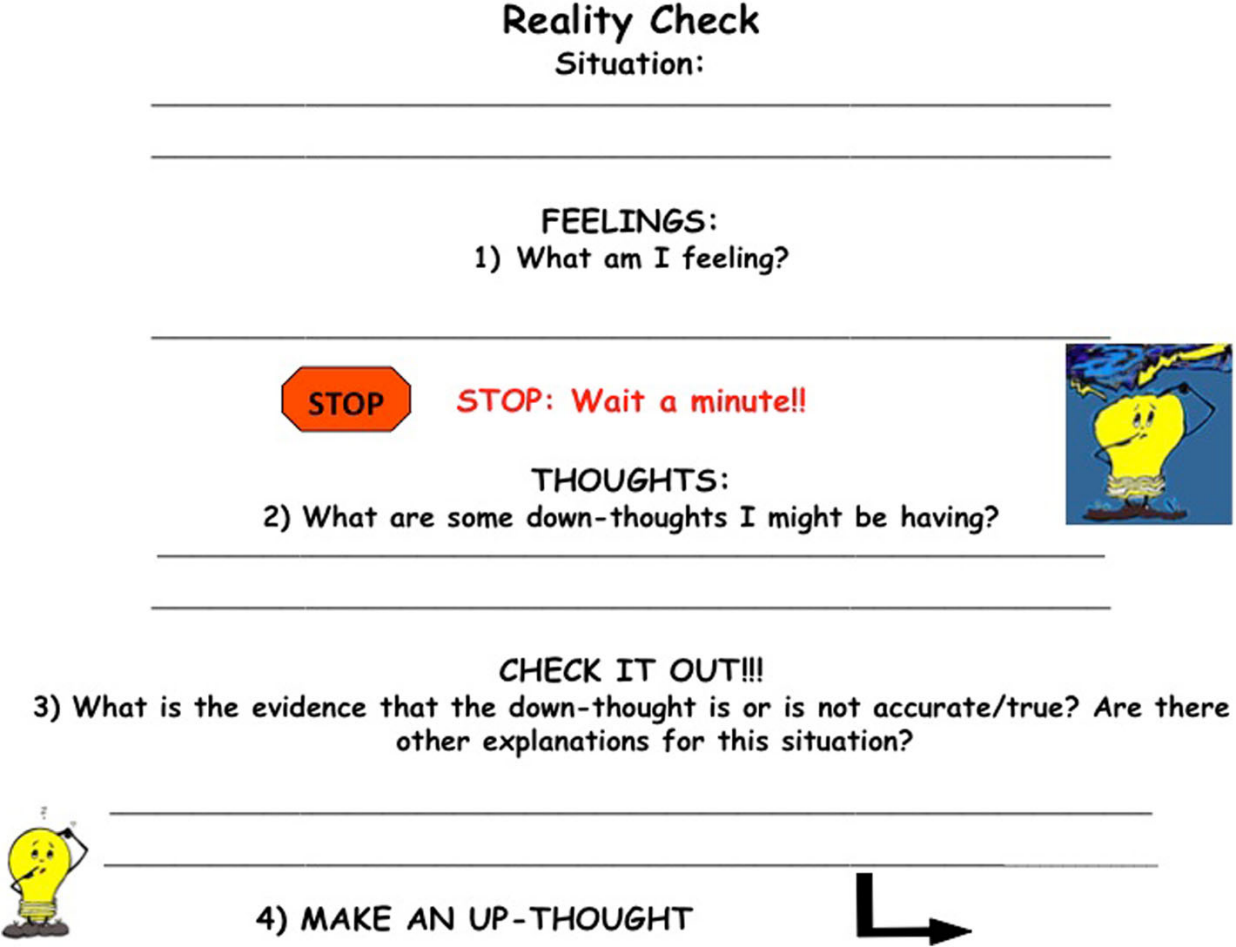
Reality Check worksheet

In the “Think Tank” module, group members are introduced to the concept of the “Reality Check,” which helps to stop the progression of down-thoughts. Through multiple activities, students learn to identify negative emotions they may be experiencing and how to stop and evaluate why they might be feeling such emotions. The steps to completing a Reality Check are provided to the group members in the form of a visual aid to facilitate the process (Fig. 7). One important aspect of being able to successfully complete a Reality Check is the ability to identify a specific down-thought and find the evidence for and against it. In another activity, group members play the role of a Reality Check Detective and use their knowledge about down-thoughts to find the evidence. The ability to provide evidence for and against the down-thought allows the group members to investigate their reaction to an event or circumstance and decide whether their reaction is an appropriate match to the situation. The reality check also serves to decelerate the automatic thought process that may lead to a downward spiral of emotions and behaviors.

**Fig. 8:**
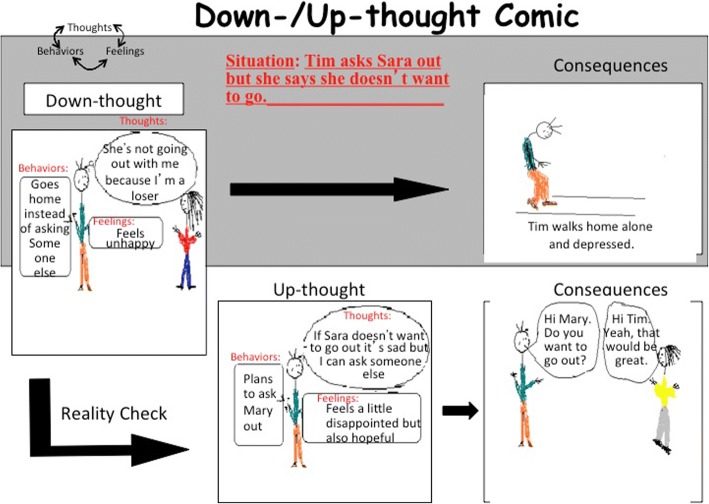
Example of the down/up-thought comic used in the program

While the bulk of the “Think Tank” module is used to practice doing reality checks, the remainder of the module focuses on how to turn down-thoughts into up-thoughts. As mentioned above, up-thoughts are defined as helpful and realistic thoughts that support our progress towards a goal. Though it is important to be able to identify a down-thought, it is equally as important to be able to turn said down-thought into an up- thought. Making a slight change in the wording of the thought can have a drastic impact on the content of the thought, as well as the connected emotions and behaviors we exhibit thereafter. For example, changing a thought from “*Nobody* likes me” to “*Some people*/NAME OF PARTICULAR PERSON like (s) me” creates some hope. In order for the change in semantics to be relevant, the group member must also be able to understand how the function of the thought is being transformed from an unproductive and limiting down-thought to a helpful and realistic up-thought. One activity that the group members partake in to assist in this process is the down/up-thought comic (Fig. 8). By drawing their own comic, group members are able to use their creativity to illustrate the possible consequence of a down-thought versus that of an up-thought. Participation in this activity allows for group members to easily see the stark contrast in the resulting behaviors that can emanate from different ways of thinking about a given situation.

#### Module 4: “Just Do It”

**Fig. 9:**
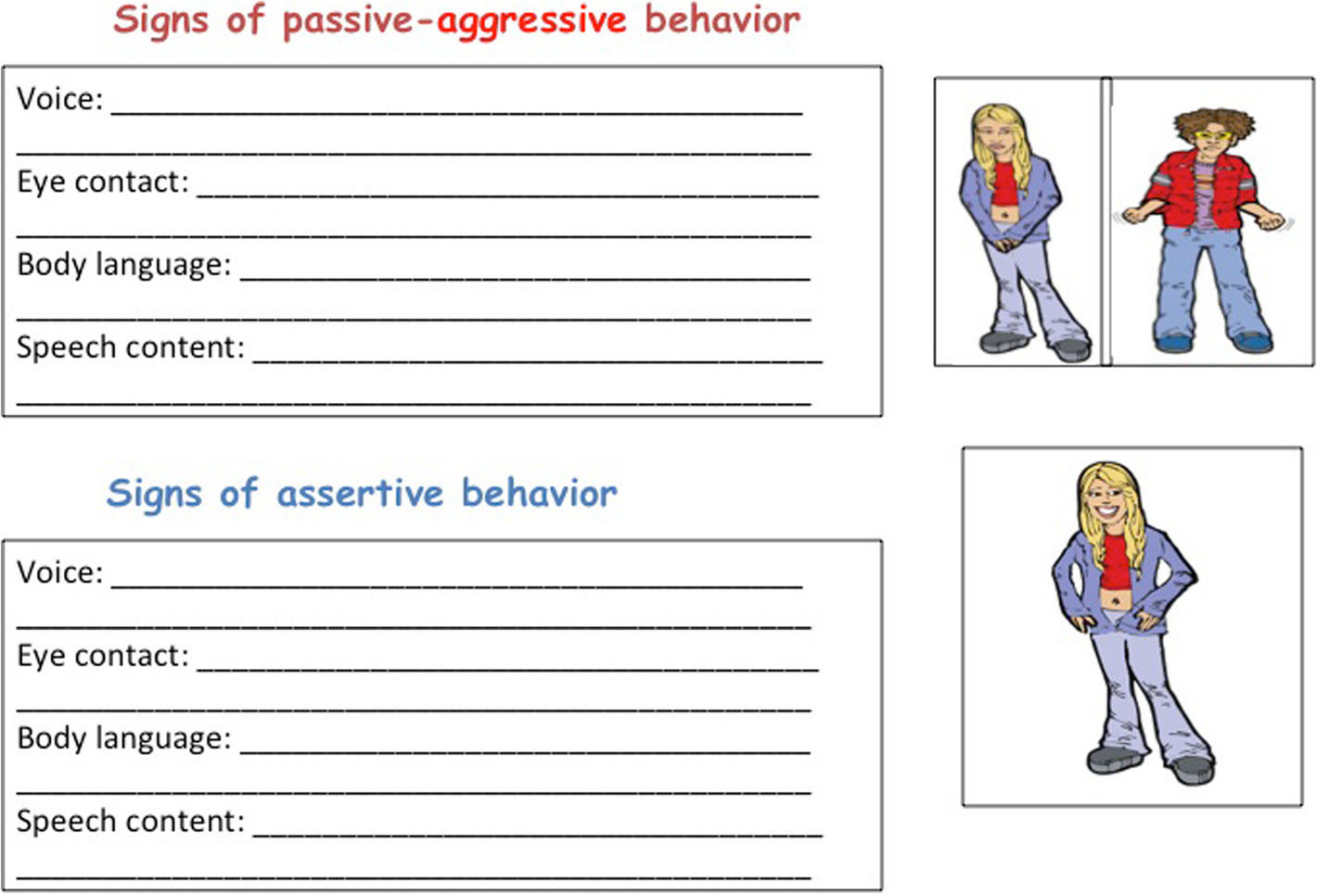
Worksheet to collect signs of passive-aggressive and assertive behavior

The last two LARS&LISA modules focus on behavior and how to implement all of the preceding content. Just Do It connects thoughts and behaviors and addresses the pros and cons of various types of behavior. Specifically, passive, aggressive, passive-aggressive, and assertive behaviors are discussed in this module. Multiple role-plays lead by the group leaders are used as a way to visually demonstrate the four types of behaviors (Fig. 9). Additionally, the role-plays allow the group members to observe and reflect on the response that each type of behavior might elicit from another person. For example, in the assertive role-play script, actors 1 and 2 make plans to go to the mall but actor 2 does not show up. Actor 1 is upset about the situation and must use assertive communication and behavior to attempt to resolve the issue.

**Fig. 10:**
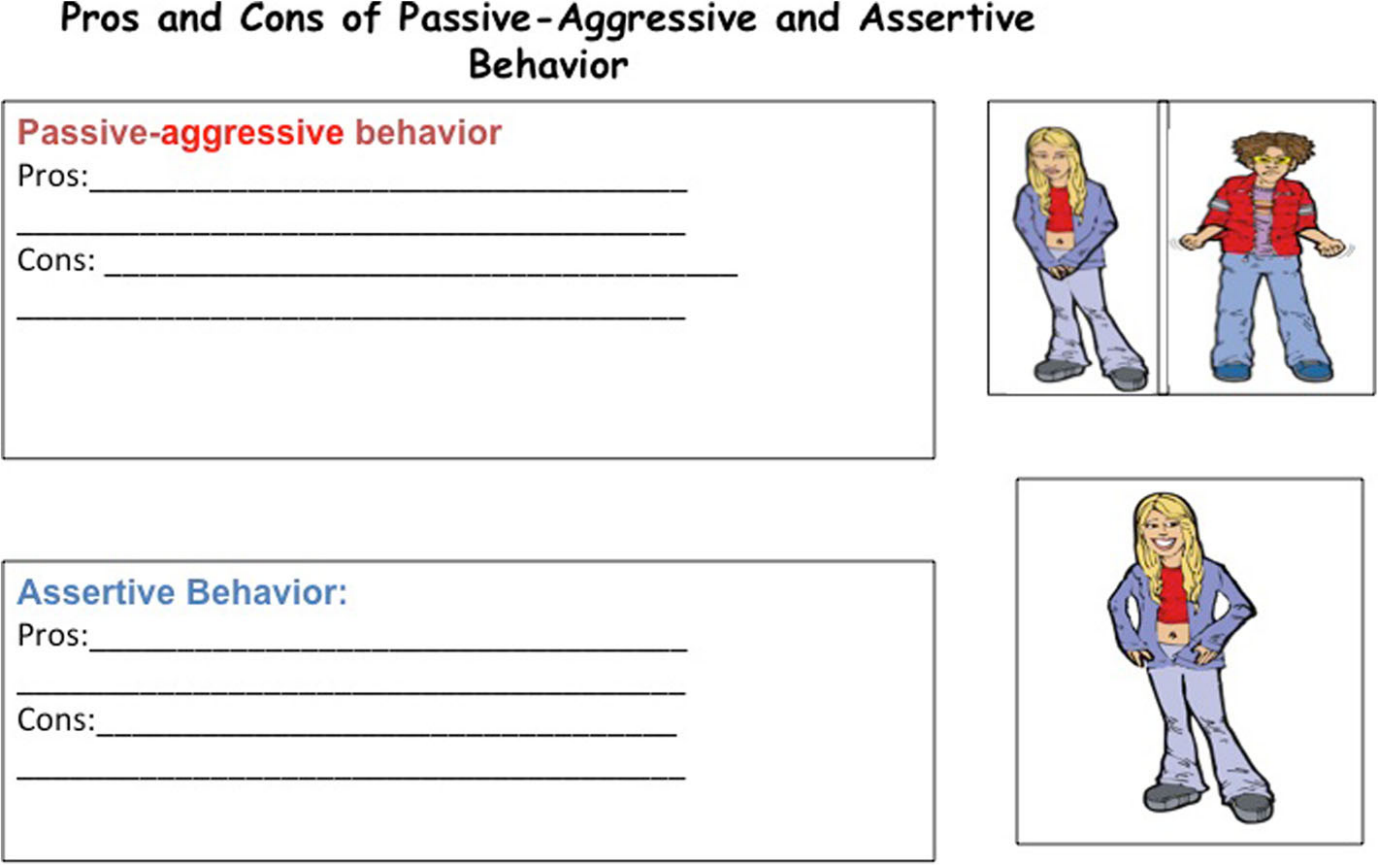
Worksheet to collect advantages and disadvantages of passive-aggressive and assertive behavior

Group members are encouraged to adopt assertive behavior over the other three types of behaviors, and therefore, more time is spent practicing and identifying signs of assertive behavior. Acting assertively allows individuals to communicate more effectively and make progress towards their goals. While assertive behavior is encouraged, it is recognized that each type of behavior has both advantages and disadvantages. Individuals have reasons for choosing to enact various behaviors, including when they opt not to be assertive. Thus, to avoid resistance while promoting assertive behavior, the pros and cons of each type of behavior are discussed (Fig. 10). Additionally, if brought up by group members, the rare circumstances when acting assertively might not be the most appropriate form of behavior are discussed and acknowledged.

Following the group leader-led role-plays and the discussions described above, group members practice assertive behavior by creating and acting out their own role-plays. In these role-plays, each group member participates as the leading actor in the same scenario twice. Repeating the role-plays offers students the opportunity to implement two to three new behaviors that were absent from their first attempt and provides them with the positive experience of seeing their behavior improve.

#### Module 5: “Making Contact”

The last module of LARS&LISA, “Making Contact,” focuses on another part of the behavior aspect of the Reversible Spiral. This module allows the group members to continue practicing assertive behavior in “real life” scenarios or situations they would likely encounter in high school. “Making Contact” goes beyond the intrapersonal impacts of displaying assertive behavior and extends to identifying ways of showing interest in others and to responding empathetically. Group members spend time thinking about when it may be important to make contact, why it is important, and how it is accomplished. Situations from meeting a new friend to applying for a job are all instances in which having the skills to make contact are necessary.

**Fig. 11:**
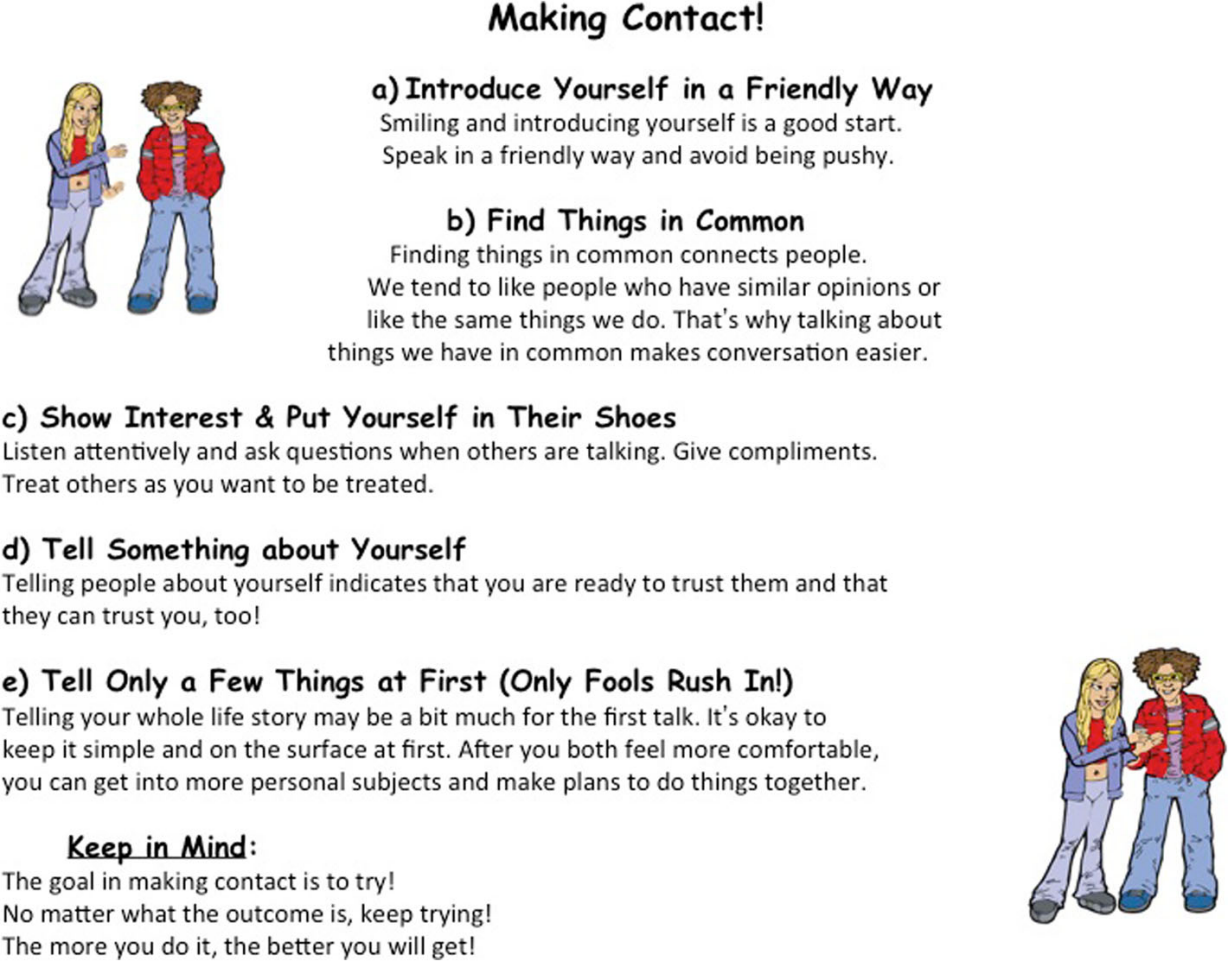
List of steps how to appropriately make contact with others

The module begins with a discussion about the importance of showing interest in others and responding empathetically. Learning how to appropriately make contact with others across settings is an important skill that most people have to implement on a daily basis (Fig. 11). Learning to communicate effectively provides opportunities to establish positive relationships with others, which is often rewarding because it can lead to companionship or support. While the possible reward for making contact could be new relationships, success is not measured by the response of others. Instead, success is measured by the person’s attempt to be assertive and communicate effectively. In the latter part of the module, group members have a chance to practice making contact using their own hypothetical scenarios.

### Structure of the sessions

As is typical for a cognitive-behavioral program, each LARS&LISA session follows the same structure (Pössel, Horn, Seemann, & Hautzinger, 2004). Common elements incorporated into every session include making a semi-circle, reviewing the group guidelines, reviewing the knowledge check, presenting the session agenda, working on the session-specific content, and providing feedback regarding participation and observance of the group guidelines at the end of the session.

The authors (Pössel, Horn, Seemann, & Hautzinger, 2004) recommend that the group sit in a semi-circle for multiple reasons. One advantage is that a semi-circle creates an open atmosphere and encourages teamwork. Associated with this, by sitting in the semi-circle, the group leaders appear less like an external authority figure and more like fellow members of the group. Further, the semi-circle allows the group leaders to alter seating assignments, including their own position in the group, as they see fit. Not only is this conducive for some of the activities, but it can also be utilized to reduce distractibility and promote the adolescents’ focus on the content.

In the first session, the group leaders introduce some general guidelines to set expectations and promote the desired atmosphere in the group (i.e., fairness, respect, teamwork, working atmosphere; Pössel, Horn, Seemann, & Hautzinger, 2004). While the group leaders should keep the overarching goals of the guidelines in mind, the adolescents are usually a good source for generating their own guidelines that will promote cohesion and collaboration in the group and allow them to participate fully without fear of judgment. For example, in some groups, having a smart phone out might not be a problem, while in other groups, it could be a good idea to collect them before the group starts. Thus, the guidelines should be set in a collaborative manner together with the adolescents. It is beneficial to go over the agreed guidelines at the beginning of the first few, if not all, sessions. This serves as a helpful reminder and also allows the group leaders and adolescents to introduce new guidelines or adapt the existing ones if necessary. This will seem like a natural process when the group leaders regularly go over the guidelines and point out what worked well in the last session and identify any issues that need to be addressed.

Another structural element of LARS&LISA is what the authors call a knowledge check (Pössel, Horn, Seemann, & Hautzinger, 2004). This is a short quiz about the content of a session that is handed out to the adolescents at the end of the session. By the next session, the group leaders correct the knowledge check and provide positive feedback (positive reinforcement) to the whole group at the beginning of the next session. The main purpose of this structural element is to assess whether the adolescents understood the material of the session and to identify how much and what parts of the content need to be reviewed. In addition, correcting the knowledge check ensures that each adolescent has the correct answers to questions on the most important elements of the content.

Next, it is recommended that the group leaders go over the agenda of the session, which includes the topics and the goals for the day (Pössel, Horn, Seemann, & Hautzinger, 2004). This provides structure for the session and informs the adolescents about what they can expect, which provides them with some sense of control. Beginning with an agenda also encourages the group members to connect the material from former sessions to what will be covered in the present session.

After working through the session-specific content, the group leaders help the members to make connections between current and past session material, as well as give feedback to the adolescents regarding their participation and observance of the group guidelines. The authors emphasize that making these connections is crucial. Thus, in the making connections portion of the session, adolescents are invited to find examples of how they used what they have learned so far in the previous sessions in their everyday life. The group members are encouraged to think about and share how applying what they learned can help them to reach their goals. According to the authors, this shows adolescents how the program can influence their everyday lives, encourages them to use the content of LARS&LISA, and maintains their motivation to actively participate. In regard to the feedback session, Pössel, Horn, Seemann, and Hautzinger (2004) express that the feedback should always be positive and reinforcing, using specific examples about what guidelines were followed when and by whom. Negative feedback is discouraged as it may provoke a reaction from the students or deter future participation.

### Groups and group leaders

In the published manual, the authors state that the ideal group size is between 8 and 12 students but they also implemented groups with fewer (*n* = 4) and more (*n* ≤ 20) adolescents (Pössel, Horn, Seemann, & Hautzinger, 2004). Further, LARS&LISA groups have been led by one, two, and even three group leaders. However, the default is to have two group leaders to implement a group as this allows:

- For small group work without leaving any group unsupervised (unsupervised small group work is usually associated with a drop in focused work, independent of the age of the group members),
- One group leader to focus on teaching the content of the program while the other one can keep the adolescents focused and/or answer questions from individual adolescents without slowing down the whole group,
- The group leaders to switch things up and take turns presenting the content, thus making it more interesting.

Pössel and his team also make suggestions about the knowledge, experience, and behavior group leaders should have and show, respectively (2004). They suggest that knowledge about cognitive and learning theory and of social skills trainings is helpful. Further, they emphasize that experience with adolescents, as well as in running groups and the dynamic of groups, is beneficial. When it comes to the actual implementation of the groups, they suggest that group leaders should be able to differentiate between knowledge-related and personal questions and call on adolescents to answer the former but rely exclusively on volunteers when it comes to the later. Further, group leaders must ensure understanding of theoretical background, be transparent about the group goals, and handle “wrong” answers appropriately by reframing and acknowledging the “correct” parts of answers. Positive reinforcement is emphasized as a means of encouraging wanted behavior, and difficult group situations should be addressed in a way that supports the development of a collaborative, positive, trusting, and work-focused group atmosphere.

Pössel et al. (2013) describe that group leaders are trained in two steps. First, future group leaders participated in a mock version of the program. Second, they study the manual, materials, and procedures and discuss their questions with their supervisors. Further, when they implement the program, they participate in weekly supervision using video recordings of each session. This serves to ensure adherence to the manual and further training of the group leaders.

## Does LARS&LISA work, for whom and when?

Multiple meta-analyses have indicated that youth depression prevention interventions are effective (e.g., Brunwasser, Gillham, & Kim, 2009; Horowitz & Garber, 2006; Merry et al., 2011; Stice, Shaw, Bohon, Marti, & Rohde, 2009). However, the effects of the programs range from small to modest, and multiple factors moderate the effects of these programs. These moderators included the type of prevention program (i.e., universal, selective, indicated), participant attributes (e.g., age, sex, race), characteristics of the intervention (e.g., duration, content), group leaders (e.g., level of training), and timing of assessments (e.g., post-intervention, follow-ups of various lengths). The effect sizes also differ depending on the type of control condition utilized. Studies comparing the effects of an intervention with a no intervention or waitlist control condition tend to find larger effect sizes, whereas studies that utilize an active or placebo control condition tend to find comparatively smaller effect sizes (Cuijpers et al., 2008). These factors involved with measuring the effects depression prevention are relevant to the evaluations of the LARS&LISA program discussed below.

When examining the effects of universal prevention, two issues that are different from the evaluation of therapy programs should be considered. First, as Pössel, Horn, and Hautzinger (2006) point out, while the lifetime incidence of subsyndromal and major depression are relatively high (Bertha & Balázs, 2013; Kessler, Petukhova, et al., 2012), the point prevalence of major depression in the general population is not high enough to examine the effects of a program by the prevention of diagnosable depression without an unrealistically high sample size. Thus, the main outcome variable in most, if not all, studies examining universal prevention is not the reduction of new cases of major depression, but the reduction or prevention of worsening of depressive symptoms. This leads to the second issue that is different in studies examining universal prevention. The majority of participants in studies examining universal prevention will show no or only very low levels of depression at baseline. Thus, contrary to therapy, which aims to reduce existing symptoms (therapy effect), the aim of universal prevention will often not be the reduction of symptoms, but the prevention of an increase of symptoms (prevention effect). Both issues should be kept in mind when reviewing the evidence to the different versions of LARS&LISA. To allow the readers to evaluate the strengths of the effects discussed below, we present effect sizes provided in the original articles. When the authors did not provide effect sizes, we calculated Hedge’s *g* based on the information provided in the original articles if possible.

The original LISA program has been evaluated in studies from two different research groups (Groen et al., 2003; Pössel et al., 2005; Pössel et al., 2008; Pössel, Horn, Groen, & Hautzinger, 2004; Pössel, Horn, & Hautzinger, 2006). The first evaluation study was conducted by the developers of the program with German eighth grade students and tested participant acceptance of LISA. More than 2/3 of the adolescents rated LISA as good or very good, and between 60.9% and 67.6% of the adolescents reported they had learned something they could use in their everyday lives (Pössel, Horn, & Hautzinger, 2003). While the authors interpreted these data as an indication that the content and skills included in LISA are acceptable to and understood by adolescents from the general population, they still made the changes outlined above to further increase the acceptability and applicability of the program.

The first evaluation study also assessed the effect of LISA on self-reported depressive symptoms (Pössel, Horn, Groen, & Hautzinger, 2004). As to be expected based on the prevention effect described above, adolescents with minimal depression scores at baseline who had participated in LISA did not experience significant increases of their depressive symptoms during the following 6 months (*g* = 0.33, 95% CI = − 0.03 to 0.69, calculated for this article). At the same time, their peers in a non-intervention control condition reported significant increases in depressive symptoms (*g* = 0.87, 95% CI = 0.42 to 1.32, calculated for this article). Consistent with these findings, the percentage of adolescents with only minimal depression scores who participated in LISA increased from baseline to 6-month follow-up from 40.7 to 64.3% indicating that a number of participants experienced a decrease in depressive symptoms, whereas percentages in the control group remained stable (37.2% vs. 37.6%). As to be expected following the therapy effect described above, adolescents with subclinical depression at baseline who participated in LISA reported a significant reduction of depressive symptoms from baseline to 6-month follow-up (*g* = − 0.41, 95% CI = − 0.74 to − 0.08, calculated for this article), whereas the depression scores of their peers in the control group did not change during the same time period (*g* = 0.14, 95% CI = − 0.24 to 0.51, calculated for this article). Consistent with these findings, the percentage of adolescents with subclinical depression participating in LISA decreased from 52.0 to 31.2%, while percentages in the control group remained stable (50.4% vs. 51.4%). Thus, in this study, LISA had a positive effect on the depressive symptoms of adolescents with minimal and subclinical levels of self-reported depressive symptoms (Pössel, Horn, Groen, & Hautzinger, 2004). Subsequent analyses using the data of the same study to calculate moderation analyses have confirmed the positive effect of LISA indicating that the program is particularly beneficial for adolescents with low global self-efficacy (Pössel et al., 2005). To be more precise, depressive symptoms in adolescents with low self-efficacy in the control group increased over time (*g* = 0.43, 95% CI = − 0.09 to 0.95) and depressive symptoms in adolescents with low self-efficacy were significantly lower in the group that participated in LISA than in the control group (*g* = 0.75, 95% CI = 0.26 to 1.24). Further, adolescents with minimal depression scores at baseline who participated in LISA reported fewer depressive symptoms than adolescents participating in an active control group (*g* = 0.50, 95% CI = 0.22 to 0.77) using an expressive writing paradigm (Pennebaker & Beal, 1986) and a non-intervention control group (*g* = 0.50, 95% CI = 0.20 to 0.80, calculated for this article) at post-intervention. Adolescents with subclinical depression at baseline who participated in LISA reported significantly lower depressive symptoms at post-intervention compared to adolescents participating in the active control group (*g* = 0.42, 95% CI = 0.12 to 0.72, calculated for this article) and the non-intervention control group (*g* = 0.47, 95% CI = 0.14 to 0.81, calculated for this article) at follow-up (Pössel, Horn, & Hautzinger, 2006).

The second evaluation study of LISA was conducted by an independent group of researchers with German seventh grade students using a non-intervention control condition for comparison (Groen et al., 2003). While Dodge’s social information processing model (1993) explains the development and maintenance of depression and aggressive behavior, this study examined the effects of LISA on both. Consistent with the prevention effect, the authors of this study found no changes in aggressive behavior in the LISA group (*g* = − 0.02, 95% CI = − 0.30 to 0.26, calculated for this article), while aggressive behavior significantly increased the control group over time (*g* = 0.23, 95% CI = 0.03 to 0.49, calculated for this article). Further, aggressive behavior was significantly lower in adolescents participating in LISA compared to control group adolescents at follow-up (*g* = − 0.42, 95% CI = 0.15 to 0.69, calculated for this article). However, Groen et al. did not find any significant changes or differences in depressive symptoms. The authors explain the lack of a significant effect of LISA on depressive symptoms in this study with the finding that there was no increase in depressive symptoms in the non-intervention control group. Considering that the participants in this study were seventh grade students (mean age = 12.50 years, *SD* = 0.63), Groen et al. propose that the lack of increase in depressive symptoms in the control group is associated with the age of the adolescents. This is consistent with epidemiological findings that rates of depression do not begin to increase dramatically until the mid-teen years (for a systematic review see Bertha & Balázs, 2013), which is beyond the mean age of the participants in Groen et al.’s study. Thus, no increase in depressive symptoms could be prevented, and therefore, no prevention effect on depressive symptoms could be observed.

A study evaluating the revised program (LARS&LISA) involving German eighth grade students indicated that the program is similarly effective in adolescents with and without comorbid anxiety symptoms and conduct problems (Pössel et al., 2008). However, analyses examining possible gender differences revealed that female adolescents benefited from participating in LARS&LISA independent of their level of depressive symptoms at baseline, while their male peers benefited if they had minimal levels of depressive symptoms at baseline, but not if they had subclinical levels of self-reported depressive symptoms (Pössel et al., 2008). To expatiate, boys with minimal depression scores at baseline in the LARS&LISA group reported significantly more severe depressive symptoms than their peers in the control group at baseline (*g* = 0.53, 95%; CI = 0.06 to 1.00), but this difference became non-significant at post-intervention (*g* = 0.40, 95%; CI = − 0.07 to 0.87) and remained non-significant at follow-up (*g* = 0.15, 95%; CI = − 0.29 to 0.60). Further, depressive symptoms in boys with minimal depression scores at baseline increased significantly more among those in the control group compared to those in the LARS&LISA group over time (*g* = 0.51, 95%, CI = 0.04 to 0.98). For girls, depressive symptoms decreased significantly over time among participants in LARS&LISA, regardless of whether they had minimal depression scores (*g* = 0.61, 95%; CI = 0.09 to 1.13) or subclinical depression at baseline (*g* = 0.52, 95%; CI = 0.08 to 0.95) Meanwhile, depressive symptoms in girls in the control group did not change over time. Further examining the gender difference, Pössel, Adelson, and Hautzinger (2011) found that knowledge about the content of LARS&LISA, but not conduct problems, might partially explain the difference. Thus, the authors suggested further examining possible gender differences in the effects of depression prevention programs in general and LARS&LISA in particular.

Besides focusing on gender differences, the purpose of another evaluation study of LARS&LISA in German eighth grade students was to examine if teachers could implement the program successfully (Wahl, Adelson, Patak, Pössel, & Hautzinger, 2014). Consistent with the previous evaluations in which prevention programs were implemented by psychologists and teachers in separated studies (Penn Resiliency Program: Gillham, Hamilton, Freres, Patton, & Gallop, 2006; Gillham et al., 2007; Resourceful Adolescent Program: Harnett & Dadds, 2004; Merry et al., 2004), the authors of this study found psychologists to be more effective group leaders than teachers. More specifically, a decrease in depressive symptoms was observed over time for girls participating in LARS&LISA when it was implemented by psychologists (*g* = − 0.36, 95% CI = − 0.68 to − 0.04, calculated for this article). However, no such effect was found in boys (*g* = 0.30, 95% CI = − 0.04 to 0.57, calculated for this article) or when LARS&LISA was implemented by teachers (girls: *g* = − 0.10, 95% CI = − 0.40 to 0.20; boys: *g* = − 0.10, 95% CI = − 0.39 to 0.19, calculated for this article). Wahl and colleagues concluded that their results, combined with previous similar findings for other prevention programs (Gillham et al., 2006, 2007; Harnett & Dadds, 2004; Merry et al., 2004), highlight a limitation to the widespread dissemination of programs like LARS&LISA, considering the likelihood of any school system hiring enough psychologists to comprehensively implement this type of prevention.

Finally, the purpose of the study using the American version of LARS&LISA (TIM&SARA) was to overcome a limitation of all previous studies examining this program and most other depression prevention programs, the lack of a structurally equivalent control condition (Pössel et al., 2013). In their meta-analysis of psychotherapy studies, Baskin, Tierney, Minami, and Wampold (2003) found similar effects for specific (e.g., cognitive-behavioral therapy) and nonspecific programs when they had structural equivalence, which they defined as being identical in regards to the number and duration of sessions, settings (group vs. individual), level of therapists’ experience, and adaptability of the therapy to the client. This raises a question regarding whether a successful prevention program has to be as complex as LARS&LISA or if simpler programs (which might also be easier to implement) can be comparably beneficial. In their study with American ninth grade students, Pössel and colleagues found that adolescents participating in TIM&SARA reported significantly lower depression scores compared to the structurally equivalent non-specific prevention program (*g* = 0.29, 95% CI 0.06 to 0.52) and a non-intervention control condition (*g* = 0.30, 95% CI 0.07 to 0.53) at 4-month follow-up. However, these differences did not remain significant as the depression scores in the non-specific prevention (8-month follow-up *g* = 0.26; CI 0.03 to 0.48; 12-month follow-up *g* = 0.34, 95% CI 0.11 to 0.56) and non-intervention control (8-month follow-up *g* = − 0.32, 95% CI − 0.10 to − 0.55; 12-month follow-up *g* = − 0.28, 95% CI − 0.05 to − 0.50) groups decreased at the later follow-ups as well.

LARS&LISA shows positive effects on aggressive behavior (Groen et al., 2003) and depressive symptoms for American (Pössel et al., 2013) and German adolescents (Pössel et al., 2005; Pössel et al., 2008; Pössel et al., 2011; Pössel, Horn, Groen, & Hautzinger, 2004; Pössel, Horn, & Hautzinger, 2006; Wahl et al., 2014). To be more precise, female adolescents benefit independent of their level of depressive symptoms while male adolescents with minimal levels of depression at baseline tend to benefit more than males with higher levels of depressive symptoms at baseline (Pössel et al., 2008). This difference is at least partially explained by knowledge of the content of LARS&LISA (Pössel et al., 2011). Further, the program benefits adolescents independent of comorbid symptoms of anxiety and conduct problems (Pössel et al., 2008), and it is superior to other programs (Pössel et al., 2013; Pössel, Horn, & Hautzinger, 2006) as long as it is implemented by psychologists (Wahl et al., 2014).

## Conclusions

Based on the last 20 years, what are the next steps in the ongoing iterative process between program development, empirical studies, and feeding the empirical evidence back into the program development? As the comparison with the structurally equivalent non-specific prevention program (Pössel et al., 2013) hints that the effects of LARS&LISA are based on specific cognitive-behavioral interventions, it seems logical to further examine the underlying mechanisms of change. Elucidating those mechanisms might allow us to (a) understand why male adolescents with elevated depressive symptoms at baseline seem not to benefit as much as other youth, (b) improve the effects of the program (for all participants), and (c) simplify the program by focusing on the parts that make LARS&LISA effective and, with that, maybe even reconsider teachers as possible group leaders. In this context, the question raised by Wahl et al. (2014) and other studies (Gillham et al., 2006, 2007; Harnett & Dadds, 2004; Merry et al., 2004) about the feasibility of wide-spread dissemination of LARS&LISA, or any similar program, if the program must be delivered by psychologists is a valid concern, and elucidating the mechanisms of change and thereby the reasons why teachers were unable to effectively implement the program is an important area for future research. Of interest in this context are some findings from previous studies. As mentioned above, Pössel et al. (2011) found that the knowledge of cognitive and social parts of LARS&LISA partially explained gender differences in the effects of the program. This raises the question of why male adolescents seem to acquire or retain less of the program’s content and how their understanding can be improved. Further, Pössel and colleagues (2003) report that immediately after the end of LISA (baseline-post comparison) female adolescents reported using their previously existing social network more often while male adolescents tended to increase their social network. Another study showed that changes in cognitive errors seem to mediate the effects of TIM&SARA (Pössel, Martin, Garber, & Hautzinger, Submitted-a). Unfortunately, in this publication, adolescent gender was not considered. Finally, in a publication to LARS&LISA, Pössel, Roane, and Hautzinger (Submitted-b) found that the program impacts frontal brain activity in male but not female adolescents while it influences depressive symptoms in female but not male adolescents. Summarized, there is some evidence that both cognitive and social elements make the program effective, but it might be that both genders benefit differently from these two aspects. Consequently, a more systematic examination of possible change mechanism by gender interactions seems necessary. Associated with this consideration, an exploration of potential gender differences in the social information processing (Dodge, 1993) of depressed adolescents might be beneficial. For example, one could speculate that males tend to be more likely to produce multiple problem-irrelevant responses on the stage of *response accessing*, while females might evaluate withdrawing behavior as more positive during the *response evaluation and selection* stage. Based on the logic of the program, role-plays should be helpful for both males and females, but the purpose and therefore the focus of the role-plays might be a crucial factor in maximizing benefits for both genders. One possible outcome of an examination of the underlying change mechanisms might be two programs that are similar but tailored to the specific needs of the gender of the participating adolescents (LARS and LISA).

An issue that many prevention researchers wrestle with is that it seems unrealistic to expect schools can implement programs for each and every problem their students might encounter. Thus, the above reported findings regarding aggressive behavior (Groen et al., 2003) are crucial, but only an incipient first step. It seems only logical that the authors of LARS&LISA currently examine the effects of the program on other outcome variables such as academic indices (grades and suspensions) and physical health (blood pressure, cortisol, and immune parameter in saliva). This also makes sense given that depression is known to be associated with academic (Humensky et al., 2010; Lawrence et al., 2016; Naicker, Galambos, Zeng, Senthilselvan, & Colman, 2013) and physical health issues later in life (for a meta-analyses, see Nicholson, Kuper, & Hemingway, 2006). Thus, findings regarding the effects of LARS&LISA on those variables might be pivotal for a potential dissemination of LARS&LISA and similar cognitive-behavioral programs.

Authors of future studies should consider the possibility of erroneous intervention effects that can result from the combination of using self-report measures of depressive symptoms and a lack of a believable placebo or active control condition. If adolescents are aware they participated in a depression prevention program, it is possible that they might answer questions regarding depressive symptoms post intervention in a socially desirable way, meaning they report fewer depressive symptoms than they actually experience. As only the participants in the prevention condition are likely to experience pressure to respond in this way, it is possible that erroneous intervention effects are observed. To avoid this problem, parent-, teacher-, or clinician-report measures of depressive symptoms should be collected in addition to self-reports. Further, as adolescents in a believable placebo or active control condition would experience a similar pressure to answer in a social desirable way, the authors of future studies might want to follow the example of Pössel et al. (2013) and to compare a prevention program with an equally believable placebo or active control program.

Obviously, there are many other topics related to cognitive-behavioral depression prevention programs in general and to LARS&LISA in particular that need to be explored. Thus, while the journey to help prevent depression in adolescents and the negative consequences associated with this serious mental problem has just begun, the last 20 years of program development and collection of empirical evidence produced a program that benefits most adolescents along with many insights that are relevant beyond this one specific program. At the present time, there is convincing evidence to support the efficacy and utility of universal prevention programs, including LARS&LISA. However, additional research is still needed to make such programs equally effective for all populations and increase the feasibility of widespread implementation.

## Abbreviations

LARS&LISA: Lust An Realistischer Sicht & Leichtigkeit Im Sozialen Alltag
TIM&SARA: Together Initiating More Socially Advantageous & Realistic Attitudes
